# Characteristics and predictors of acute and chronic post-COVID syndrome: A systematic review and meta-analysis

**DOI:** 10.1016/j.eclinm.2021.100899

**Published:** 2021-05-24

**Authors:** Fahad M Iqbal, Kyle Lam, Viknesh Sounderajah, Jonathan M Clarke, Hutan Ashrafian, Ara Darzi

**Affiliations:** a Department of Surgery and Cancer, Imperial College London, St Mary's Hospital, London W2 1NY, UK; b Institute of Global Health Innovation, Faculty Building, South Kensington Campus, Kensington SW7 2AZ, London, UK; c EPSRC Centre for Mathematics of Precision Healthcare, Imperial College London, London SW7 2AZ, UK

**Keywords:** Post covid syndrome, Long covid, SARS-CoV 2, COVID-19, Systematic review, Coronavirus

## Abstract

**Background:**

A significant proportion of individuals experience lingering and debilitating symptoms following acute COVID-19 infection. The National Institute for Health and Care Excellence (NICE) have coined the persistent cluster of symptoms as post-COVID syndrome. This has been further sub-categorised into acute post-COVID syndrome for symptoms persisting three weeks beyond initial infection and chronic post-COVID syndrome for symptoms persisting beyond twelve weeks. The aim of this review was to detail the prevalence of clinical features and identify potential predictors for acute and chronic post-COVID syndrome.

**Methods:**

A systematic literature search, with no language restrictions, was performed to identify studies detailing characteristics and outcomes related to survivorship of post-COVID syndrome. The last search was performed on 6 March 2021 and all pre-dating published articles included. A means of proportion meta-analysis was performed to quantify characteristics of acute and chronic post-COVID syndrome. Study quality was assessed with a specific risk of bias tool. PROSPERO Registration: CRD42020222855

**Findings:**

A total of 43 studies met the eligibility criteria; of which, 38 allowed for meta-analysis. Fatigue and dyspnoea were the most prevalent symptoms in acute post-COVID (0·37 and 0·35) and fatigue and sleep disturbance in chronic post-COVID syndrome (0·48 and 0·44), respectively. The available evidence is generally of poor quality, with considerable risk of bias, and are of observational design.

**Interpretation:**

In conclusion, this review highlights that flaws in data capture and interpretation, noted in the uncertainty within our meta-analysis, affect the applicability of current knowledge. Policy makers and researchers must focus on understanding the impact of this condition on individuals and society with appropriate funding initiatives and global collaborative research.

**Panel: Research in context**

**Evidence before this study**

The emergence of post-COVID syndrome, in which recovering SARS-CoV-2 patients suffer from persistent symptoms extending several months beyond their initial diagnosis is gaining increasing recognition. However, there is a need for a greater understanding for diagnosis and management strategies. We searched Ovid in Medline, EMBASE, health management information consortium (HMIC), and PsycINFO databases without language restriction. The search was conducted in March 2021 using a list of terms relating to COVID-19 and persistent symptoms. Studies were included if they focussed on describing ‘long-COVID’ or ‘post-COVID syndrome’, the incidence of reported symptoms or predictors. Studies detailing a follow-up period shorter than 21 days; case series and articles focussing on other non-COVID-19 related conditions were excluded.

**Added value of this study**

To the best of our knowledge, this is the first systematic review and meta-analysis describing symptom prevalence and predictors for acute and chronic subtypes of post-COVID syndrome. However, during the process, several limitations of current literature surfaced. The significant heterogeneity in the field limits clinical applicability and high-quality evidence are urgently needed.

**Implications of all available evidence**

This review highlights that flaws in data capture and interpretation, noted in the uncertainty within our meta-analysis, affect the applicability of current knowledge. Moreover, the majority of studies displayed significant risk of bias, were typically of observational design, based within a limited number of countries and of inconsistent methodologies. There is an urgent need for global collaboration and recruitment into COVID-19 trials to tackle this.

Alt-text: Unlabelled box

## 1 Introduction

As of 4^th^ February 2021, SARS-CoV-2 has infected over 70 million individuals globally and has directly attributed to over 1.6 million deaths [1]. While hospitals continue to grapple with the challenges of acute COVID-19, there is evidence to suggest the emergence of an associated secondary syndrome, labelled as either post-COVID or long-COVID syndrome, in which recovering SARS-CoV-2 patients suffer from persistent and, often, debilitating symptoms extending several months past their initial diagnosis [2], [3], [4].

In contrast to the scientific community's rapidly developing understanding of acute SARS-CoV-2 infection, characterisation of post-COVID syndrome remains sparse. It is suggested that upwards of 20% of SARS-CoV-2 positive individuals go on to develop post-COVID syndrome [5]. Its inception stems from a collective created through patients sharing a more complex course of recovery from their acute illness on social media platforms [6]. This was given further traction with healthcare professionals recovering and sharing similar experiences; it has enveloped to incorporate broader patient perspectives of recovery, extending beyond a negative test result for COVID-19, encompassing a cohort of individuals who did not require hospitalisation but suffer morbidity [7,8]. As such, there is an urgent medical, financial and societal need to understand the survivorship burden associated with this phenomenon [9], [10], [11].

Of note, there is a particular lack of understanding as to whether post-COVID syndrome constitutes a singular disease process. It has been suggested that the post-COVID syndrome may be characterised into either an acute or chronic subtype, depending on whether symptoms extend beyond 12 weeks following initial diagnosis [2,12]. However, it is not currently understood as to whether chronic post-COVID is either an extension of acute post-COVID or is a separate disease subtype that carries a distinct risk profile. Clearly delineating the clinical features between post-COVID subtypes could prove to be a crucial step in (i) empowering clinicians to accurately diagnose post-COVID in the patients that they manage in both primary and secondary care settings, (ii) counselling patients on how to manage their particular syndrome subtypes as well as (iii) ensuring appropriate resource allocation in order to cater for the specific health and social care needs associated with each subtype cohort. Moreover, these goals could be further supplemented by the prospective identification of patients who are at highest risk of developing post-COVID syndrome of any description, who may benefit from enhanced surveillance programmes upon discharge from hospital.

As such, the primary aim for this study aims to characterise the clinical features between acute and chronic post-COVID syndrome. The secondary aim is to identify predictors for post-COVID syndrome, irrespective of subtype, in order to understand the risk factors and the acute clinical course that is associated with syndrome development.

## 2 Methods

### 2.1 Design

This systematic review was conducted in accordance to the Preferred Reporting Items for Systematic Reviews and Meta-analyses (PRISMA) guidelines [13]. The review was registered at the International Prospective Register of Systematic Reviews (PROSPERO ID: CRD42020222855). .

### 2.2 Research questions

This review sought to answer the following questions:

(1) What are the clinical features associated with acute and chronic post-COVID syndrome?
(2) Which features predict the development of post-COVID syndrome?

### 2.3 Search Strategy and databases

A systematic search, with expert librarian support, was performed using electronic databases through Ovid in Medline, EMBASE, health management information consortium (HMIC), and PsycINFO databases without language restriction. The search was conducted using a list of terms relating to COVID-19 and persistent symptoms; the complete search strategy is available in Appendix 1. Further studies not captured by the search were identified through bibliometric cross-referencing. Grey literature was additionally searched.

All identified studies were uploaded to Covidence (Melbourne, Australia), a Cochrane supported systematic review package tool [14]. Initial screening was conducted by two investigators (FI and KL) to determine if the eligibility criteria were met. Discrepancies resolved by discussion. Studies meeting the inclusion criteria underwent full-text screening; supplemental references were scrutinised for additional relevant articles.

### 2.4 Study selection criteria and outcome measures

The inclusion criteria for study selection were focussed on studies describing ‘long-COVID’ or ‘post-COVID syndrome’, the incidence of reported symptoms and predictors. The last search was performed in March 2021. No language restrictions were placed.

Given the rapidly expanding literature surrounding COVID-19, a wide range of publications were included, (e.g., feature articles). Studies detailing a follow-up period shorter than 21 days; case series and articles focussing on other non-COVID-19 related conditions were excluded.

### 2.5 Data extraction

Outcome measures were the prevalence of symptoms indicative of acute and chronic post-COVID syndrome.

All included study characteristics and outcome measures were independently extracted by two investigators (FI and KL) with consensus achieved. All full text reports of studies identified as potentially eligible after title and abstract review were obtained for further review.

### 2.6 Quality assessment (risk of bias)

Risk of bias was assessed using a validated quality assessment checklist for prevalence studies [15]. This consists of ten domains for assessing internal (e.g., methods for data collection, clear case definition, reliability, duration of follow-up) and external validity (e.g., representation of sample population, selection of population, response rate); and an additional cumulative risk of bias for the assessed study. Quality assessment was assessed by one reviewer (FI) and validated by a second (KL).

### 2.7 Data analysis

We characterised studies describing symptom clusters with a follow-up period of 12 weeks or more into chronic post-COVID syndrome and studies detailing a follow-up period shorter than 12 weeks as acute post-COVID, in keeping with the definitions by the National Institute for Health and Care Excellence (NICE) [12].

A meta-analysis of proportions was performed in RStudio version 3.6.3 (R Studio, Boston, MA, USA using the metaphor package and metaprop command (Appendix 2) [16]. Forest plots were generated for all included studies. Heterogeneity was assessed with the I^2^ statistic. We considered a value less than 30% as low heterogeneity, between 30-60% moderate, and over 60% as high.

Chest pain and chest tightness were grouped into one variable, given their close clinical relationship [17]. Halpin et al [18]. represented an intensive care and non-intensive care cohort. Carvalho-Schneider et al [19]. reported repeated outcomes at days 30 and 60. Therefore, separate cohorts within these papers have been displayed.

### 2.8 Funding

No funding was received for this study; all authors had access to the data and decided to submit for publication.

## 3 Results

### 3.1 Study characteristics

**Fig. 1:**
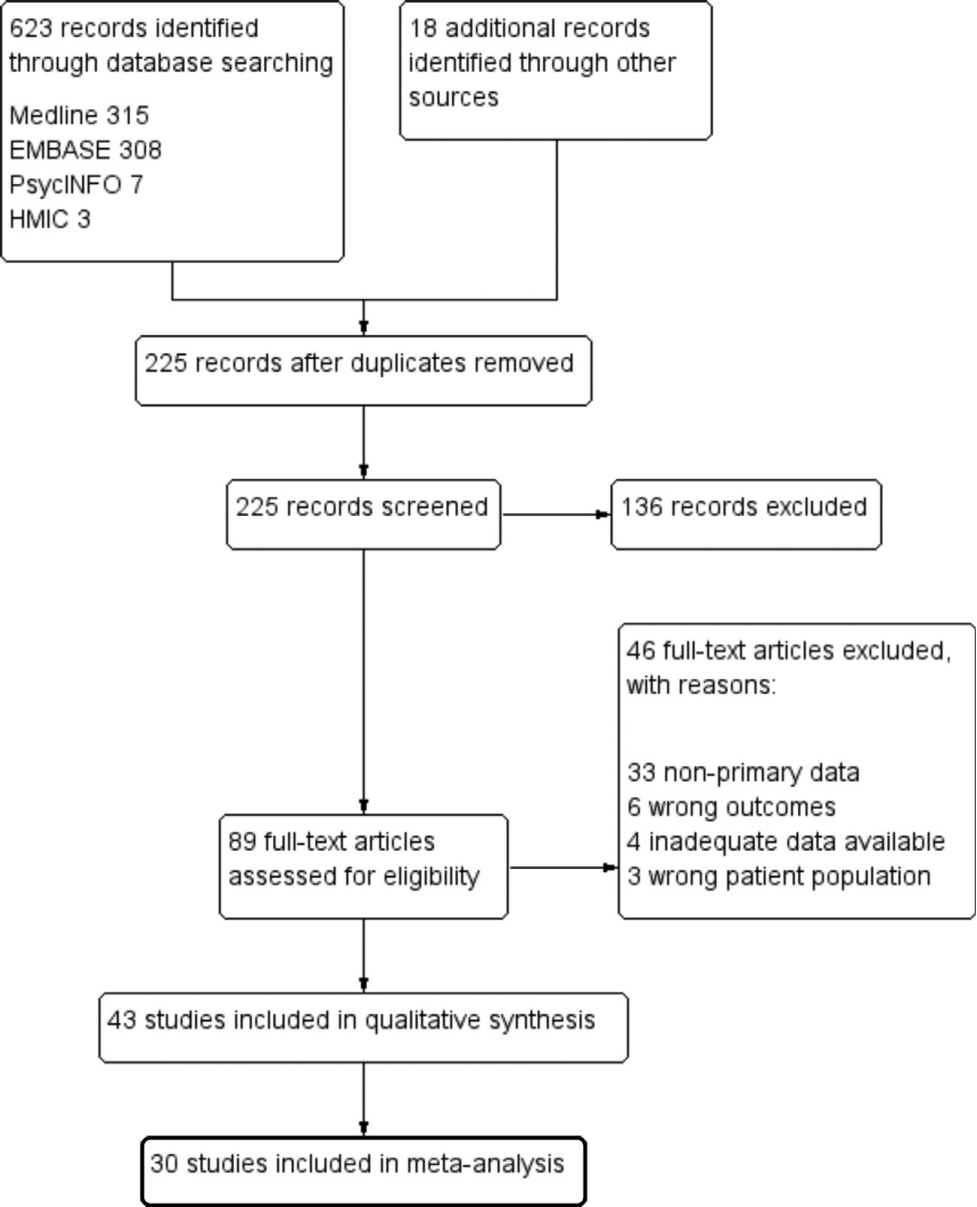
Study selection.

A total of 623 citations were retrieved through literature searches. An additional 18 articles were found from bibliography cross-referencing. Full text review was performed for 89 articles with 43 meeting the inclusion criteria for analysis, of which, 30 allowed for meta-analysis. Studies were conducted in 18 countries most of which are deemed as high-income. Included studies were observational in design with a mixture of previously hospitalised and non-hospitalised individuals recruited into the trials; the characteristics are shown in Tables 1 and 2. A PRISMA flow diagram can be seen in Figure 1.

**Table 1:** Characteristics of included studies.

Study	Country	Study type	COVID-19 status	Sampling	Risk of Bias
Arnold et al. [45]	UK	Cohort	RT-PCR confirmed cases or clinic-radiological diagnosis.	Previously hospitalised	
Bongiovanni et al. [21]	Italy	Cohort	RT-PCR confirmed cases	Previously hospitalised	
Carfi et al. [46]	Italy	Cohort	RT-PCR confirmed cases	Previously hospitalised	
Carvalho-Schneider et al. [26]	France	Cohort	RT-PCR confirmed cases	Previously hospitalised	
Daynes et al. [40]	UK	Cohort	RT-PCR confirmed cases or suspected ventilated cases	Previously hospitalised	
D'Cruz et al. [31]	UK	Cohort	RT-PCR confirmed cases	Previously hospitalised	
Garrigues et al. [39]	France	Cohort	RT-PCR confirmed cases or CT findings.	Previously hospitalised	
Halpin et al. [18]	UK	Cross sectional	RT-PCR confirmed cases	Previously hospitalised	
Jacobs et al. [24]	USA	Cohort	RT-PCR confirmed cases	Previously hospitalised	
Liu et al. [25]	China	Cross sectional	RT-PCR confirmed cases	Previously hospitalised	
Liu, Zhang et al. [47]	China	Cohort	RT-PCR confirmed cases	Previously hospitalised	-
Mandal et al. [23]	UK	Cross sectional	RT-PCR confirmed cases	Previously hospitalised	
Pellaud et al. [48]	Switzerland	Cohort	RT-PCR confirmed cases	Previously hospitalised	
Rahmani et al. [22]	Iran	Cohort	RT-PCR confirmed cases or CT findings	Previously hospitalised	
Rosales-Castillo et al. [49]	Spain	Cohort	RT-PCR confirmed cases	Previously hospitalised	
Sonnweber et al. [50]	Austria	Cohort	RT-PCR confirmed cases	Previously hospitalised	
Taboada et al. [51]	Spain	Cohort	RT-PCR confirmed cases in intensive care	Previously hospitalised	
Tomasoni et al. [52]	Italy	Cohort	RT-PCR confirmed cases or CT findings	Previously hospitalised	
Venturelli et al	Italy	Cohort	RT-PCR or serologically confirmed and suspected cases	Previously hospitalised	
Wang et al. [53]	China	Cohort	RT-PCR confirmed cases	Previously hospitalised	
Kingstone et al. [43]	UK	Qualitative	RT-PCR confirmed cases and persistent symptoms in suspected	NS	-
Sollini et al. [20]	Italy	Cohort	Persisting symptoms for >30 days in recovered cases	NS	
Blair et al. [54]	USA	Cohort	RT-PCR confirmed cases	Non-hospitalised	
Boscolo-Rizzo et al. [55]	Italy	Cross sectional	RT-PCR confirmed cases	Non-hospitalised	
Brandao Neto et al. [56]	Brazil	Cohort	RT-PCR confirmed cases	Non-hospitalised	
Chiesa-Estomba et al. [57]	Belgium, France, Spain	Cohort	RT-PCR confirmed cases	Non-hospitalised	
Fjaeldstad et al. [58]	Denmark	Cross sectional	RT-PCR confirmed cases or suspected cases	Non-hospitalised	
Lovato et al. [59]	Italy	Cross sectional	RT-PCR confirmed cases	Non-hospitalised	
Petersen et al. [41]	Faroe Islands	Cross sectional	RT-PCR confirmed cases	Non-hospitalised	
Stavem et al. [30]	Norway	Cross sectional	RT-PCR confirmed cases	Non-hospitalised	
Vaes et al. [60]	The Netherlands & Belgium	Cross sectional	RT-PCR confirmed and suspected cases	Non-hospitalised	
Villarreal et al. [61]	Spain	Cohort	RT-PCR confirmed cases	Non-hospitalised	
Darley et al. [62]	Australia	Cohort	RT-PCR confirmed cases	Mixed	
Goertz et al. [28]	The Netherlands & Belgium	Cross sectional	RT-PCR confirmed cases or suspected cases	Mixed	
Hopkins et al. [63]	UK	Cross sectional	RT-PCR or serologically confirmed cases and suspected cases	Mixed	
Islam et al. [64]	Bangladesh	Cross sectional	NS	Mixed	
Jacobson et al. [29]	USA	Cross sectional	RT-PCR confirmed cases	Mixed	
Lampl et al. [65]	Germany	Cohort	RT-PCR confirmed cases	Mixed	
Mazza et al. [66]	Italy	Cohort	RT-PCR confirmed cases	Mixed	
Poncet-Megemont et al. [67]	France	Cohort	RT-PCR confirmed cases or CT findings	Mixed	
Puntmann et al. [33]	Germany	Cohort	RT-PCR confirmed cases	Mixed	
Townsend et al. [68]	UK	Cross sectional	RT-PCR confirmed cases	Mixed	
Townsend et al. [69]	UK	Cross sectional	RT-PCR confirmed cases	Mixed	
Vaira et al. [70]	Italy	Cohort	RT-PCR confirmed cases	Mixed	
van den Borst et al. [42]	The Netherlands	Cohort	RT-PCR confirmed cases and community suspected cases	Mixed	

low; medium;  high

**Table 2:** Summary of study data.

Study	N	Mean Age, SD (y)	Female (%)	BAME (%)	Mean BMI, SD (kg/m^2^)	Common comorbidities	Follow-up timepoint	Data collected
Arnold et al.[45]	110	60 (46-73)*	44	20·9	32·1	Chronic lung disease, hypertension, DM, CHD	84 days from admission	Symptom reporting at follow-up clinic, SF-36, WEMWBS
Blair et al.[54]	118	56 (50-63)*	52·5	57·6	30 (26-30)*	Hypertension, asthma, DM, COPD	28-60 days	Self-reported symptom questionnaires
Bongiovanni et al.[21]	125	65·7	NS	NS	NS	NS	19·9 days from discharge	IES-R; PCL-5; ZSDS; BDI-13; STAI; MOS; WHIIRS; OCI scales
Boscolo-Rizzo et al.[55]	187	56 (20-89)*	55·1	NS	NS	NS	28 days from diagnosis	Self-reported symptom questionnaires: ARTIQ, SNOT-22
Brandao Neto et al.[56]	143	37·7	64·7	NS	NS	Hypertension, DM, asthma	76 (66-88)* days	Self-reported symptom questionnaires
Carfi et al.[46]	143	56·5 (14·6)	37·1	NS	26·3 (4·4)	Hypertension, Thyroid disorder, Immune disorders, COPD	60·3 (13·6) days^¥^ since symptom onset 36·1 (12·9)^¥^ days since discharge	Demographics, Covid characteristics, symptom, EuroQoL collected at outpatient visits.
Carvalho-Schneider et al.[26]	130	49 (15)	55·8	NS	NS	obesity, COPD, CKD, CHD, DM, immune disorder	30 & 60 days	EHR/phone call collected demographic & symptom data
Chiesa-Estomba et al.[57]	121	41	63·5	NS	NS	NS	47 (30-71)* days from diagnosis	sQOD-NS; self reporting symptoms
Darley et al.[62]	78	47 (16)	34·6	NS	NS	Hypertension, asthma	69 (64-83)* days from diagnosis	Self-reported symptom questionnaires
Daynes et al.[40]	131	60 (14)	41·2	NS	NS	Asthma, COPD	32 (18) days^¥^	Phone call for demographics, CAT, HADS anxiety & depression, FACIT, symptom questionnaires
D'Cruz et al.[31]	119	58·7 (14·4)^¥^	38	70	30·0 (25·9–35·2)*	CHD, COPD, CKD	61 (51–67) days from discharge	Self-reported symptom questionnaires
Fjaeldstad et al.[58]	109	39·4	79	NS	NS	NS	30 days from symptom onset	Self-reported symptom questionnaires
Garrigues et al.[39]	120	63·2 (15·7)	37·5	NS	29·2% normal/underweight 47·5% ≥ overweight	DM, hypertension	110·9 (11·1) days^¥^ following admission	Phone call collected mMRC and EuroQoL questionnaires
Goertz et al.[28]	2113	47 (39-54)*	85	NS	25 (23-29)*	NS	79 (17) days^¥^ since symptom onset	Demographics, online symptom questionnaires from two long-COVID Facebook groups
Halpin et al.[18]	100	70·5 (20-93)*† 58·5 (34-84)*‡	48·5 † 40·6‡	10·3† 34·4‡	36·8% overweight† 17·6% obese† 33·3% overweight‡ 40% Obese‡	asthma, COPD, CKD, DM	48 (10·3) days^¥^	Phone call collected symptom questionnaires, EuroQoL, demographics.
Hopkins et al.[63]	434	NS	74·9	NS	NS	NS	6 months	Self-reported online questionnaires
Islam et al.[64]	1002	34·7 (13·9(	42·1	NS	47·3% obese	DM, hypertension, CHD, malignancy, asthma	NS	Self-reported questionnaires
Jacobs et al.[24]	183	57 (48-68)*	38·5	45·9	30 (27·3-33·5)*	DM, hypertension, CHD, asthma, hyperlipidaemia	35 (± 5) days from hospital discharge	Email or telephone collected symptom questionnaires
Jacobson et al.[29]	118	43·4 (14·4)	46·6	63·6	30·4 (6·3)	NS	119·3 (33)^¥^ days from diagnosis	Symptom reporting at follow-up clinic
Kingstone et al.[43]	24	43·2	79·1	0	NS	Asthma, IBD	3-4 months - not explicitly stated.	Semi-structured interviews
Lampl et al.[65]	419	44 (30-57)*	56·6	NS	16·7% obese	NS	42 days after symptom onset	Phone call collected symptom questionnaires
Liu et al.[25]	675	55 (41-66)*	53	NS	NS	NS	37 days from discharge	GAD-7; PHQ-9; PCL-5; self-reported symptom questionnaires
Liu, Zhang et al.[47]	149	43 (36-56)*	55	NS	NS	Hypertension	21 days from discharge	CT-imaging
Lovato et al.[59]	121	46·7	59·5	NS	NS	NS	38 (3)^¥^ days from diagnosis	Phone call collected symptom questionnaires
Mandal et al.[23]	384	59·9	38	43	NS	Hypertension, DM, Asthma, COPD, CKD, CHD	54 (57-59) days*	Demographics, biochemistry, imaging; in person or telephone collected follow-up data (symptom, PHQ-2 questionnaire)
Mazza et al.[66]	402	57·8 (13·3)	34·3	NS	NS	NS	31 (16)^¥^ days from discharge	IES-R; PCL-5; ZSDS; BDI-13; STAI; MOS; WHIIRS; OCI scales
Pellaud et al.[48]	196	70 (60-80)*	39	20·9% obese	NS	DM, OSA, COPD, CHD, hypertension, cancer	30 days from symptom onset	Telephone call/EHR collected data.
Petersen et al.[41]	180	39·9 (19·4)	54·5	NS	NS	Asthma, DM, Hypertension, COPD	125 (18) days^¥^	Telephones & interview collected demographics, baseline & follow-up symptoms, mMRC scale
Poncet-Megemont et al.[67]	139	48·5 (15·3)	62·6	NS	NS	NS	79 (17)^¥^ days from symptom onset	Self-reported symptom questionnaires/semi-structured interviews.
Puntmann et al.[33]	100	49 (14)	47	NS	25 (23-28)*	Hypertension, DM, COPD, asthma, CHD	71 (64-92) days from diagnosis*	Demographics, Cardiac MRI data, hs-CRP, hs-TnT, NT-proBNP
Rahmani et al.[22]	176	60 (14)	46·9	NS	26 ± 4* moderate disease 27 ± 4* severe disease	Hypertension, CHD, DM	56 days from discharge	Phone call collected symptom questionnaires.
Rosales-Castillo et al.[49]	118	60·2 (15·1)	44·1	NS	29·7 (15·1)	Hypercholesterolaemia, DM, COPD, CHD, hypertension	50·8 (6.02)^¥^ days from discharge	Specialist discussion at follow-up
Stavem et al.[30]	458	4.6	56	NS	26·9 (5·2)	DM, asthma, arthrosis, COPD, CHD	117·5 (41-200)* days from diagnosis	Self-reported symptom questionnaires.
Sollini et al.[20]	10	58	30	NS	NS	NS	NS	PET/CT results, demographics.
Sonnweber et al.[50]	135	57 (14)	43	NS	26 (5)	CHD, hypertension, COPD, asthma, DM	100 days from diagnosis	Self-reported symptom questionnaires, mMRC scores, clinical review at follow up visits.
Taboada et al.[51]	91	65·5 (10·4)	35·2	NS	NS	Hypertension, hypercholesterolaemia, DM, asthma	6 months	Interview collected data
Tomasoni et al.[52]	105	55 (43-65)*	27	NS	NS	NS	46 (43-48) days* from discharge	Self-reported symptom questionnaire.
Townsend et al.[68]	128	49·5	53·9	NS	28·7	NS	72 (62-87) days*	Outpatient appointment, demographics, biochemistry, covid characteristics, symptom questionnaires (CFQ-11)
Townsend et al.[69]	153	50·4 (12.8)	42·5	24·8	NS	NS	75 (62-117)* days from diagnosis	Self-reported symptom questionnaires
Vaes et al.[60]	1837	47 (38-54)*	86·1	NS	25·1	NS	79 (17)^¥^ days from symptom onset	Self-reported symptom diaries and questionnaires.
Vaira et al.[70]	138	50·7	51·2 (8·8)	29% obese	NS	Cardiovascular, pulmonary disorder, DM	60 days from symptom onset	self-reported symptoms; CCCRC test
van den Borst et al.[42]	124	59 (14)	40	NS	NS	asthma, COPD, CHD, hypertension	10·0 (1·7) weeks^¥^ since discharge	Demographics, imaging, laboratory results, mMRC scale, CFS, SF-36, TICCS, PTSS, IES-R, CFQ, HADS questionnaires
Venturelli et al	767	63 (13·6)	32·9	22·4% obese	NS	Hypertension, CHD< DM, COPD	81 (66-106)* days from discharge	Self-reported symptom questionnaires
Villarreal et al.[61]	230	43 (18-62)*	85	NS	NS	NS	28 days from symptom onset	VAS symptom scales
Wang et al.[53]	131	49 (36-62)*	55	NS	NS	hypertension	28 days from discharge	Self-reported symptom questionnaires

BAME: Black Asian Minority Ethnic; BMI: body mass index

⁎ median (range); ¥ mean (SD)

† for ward patients

‡ for intensive care patients; NS: not specified; COPD: chronic obstructive pulmonary disease; DM: diabetes mellitus; CKD: chronic kidney disease; CHD: coronary heart disease; IBD: inflammatory bowel disease; hs-CRP: highly sensitive c-reactive protein; hs-TnT: highly sensitive troponin T; NT-proBNP: of N-terminal pro-brain natriuretic peptide; mMRC: modified medical research council dyspnoea scale; CFQ-11: Chalder Fatigue Score; EHR: electronic health records; HADS: Hospital anxiety and Depression Scale; FACIT: Functional assessment of chronic illness therapy; CAT: COPD assessment test; IES-R: Impact of Event Scale-Revised; TICS: Telephone Interview of cognitive status; CFS: Cognitive Failure Questionnaire; PTSS: Post traumatic stress syndrome; NCSI: Nijmegen Clinical Screening Instrument; CCCRC: Connecticut Chemosensory Clinical Research Center orthonasal olfaction test; ARTIQ: acute respiratory tract infection questionnaire; WEMWBS: Warwick-Edinburgh Mental Wellbeing Scales

### 3.2 Clinical features

**Fig. 2:**
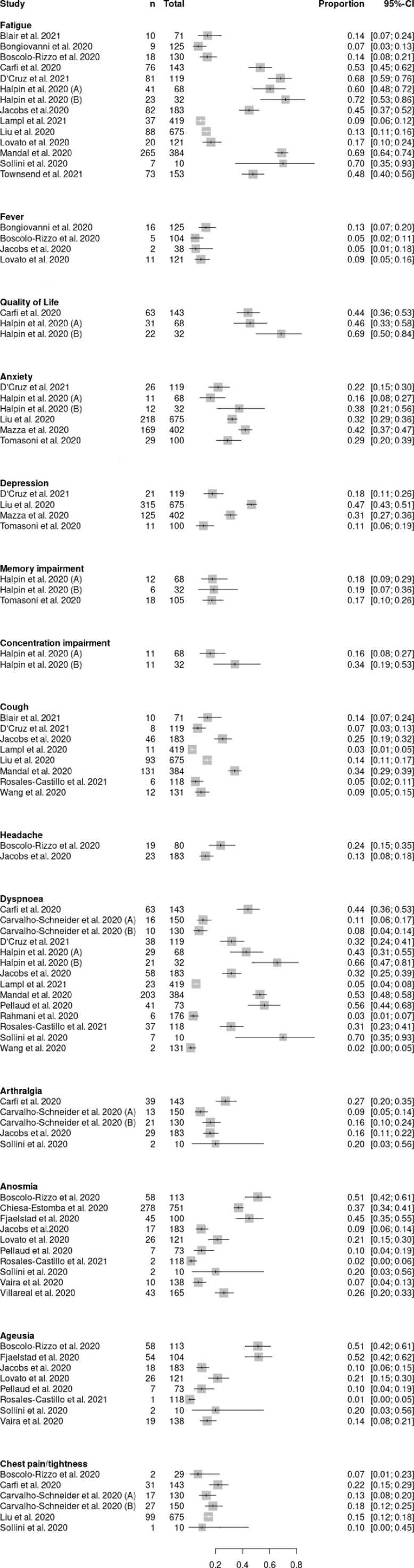
Forest plot of studies describing clinical features in acute post-COVID syndrome.

**Fig. 3:**
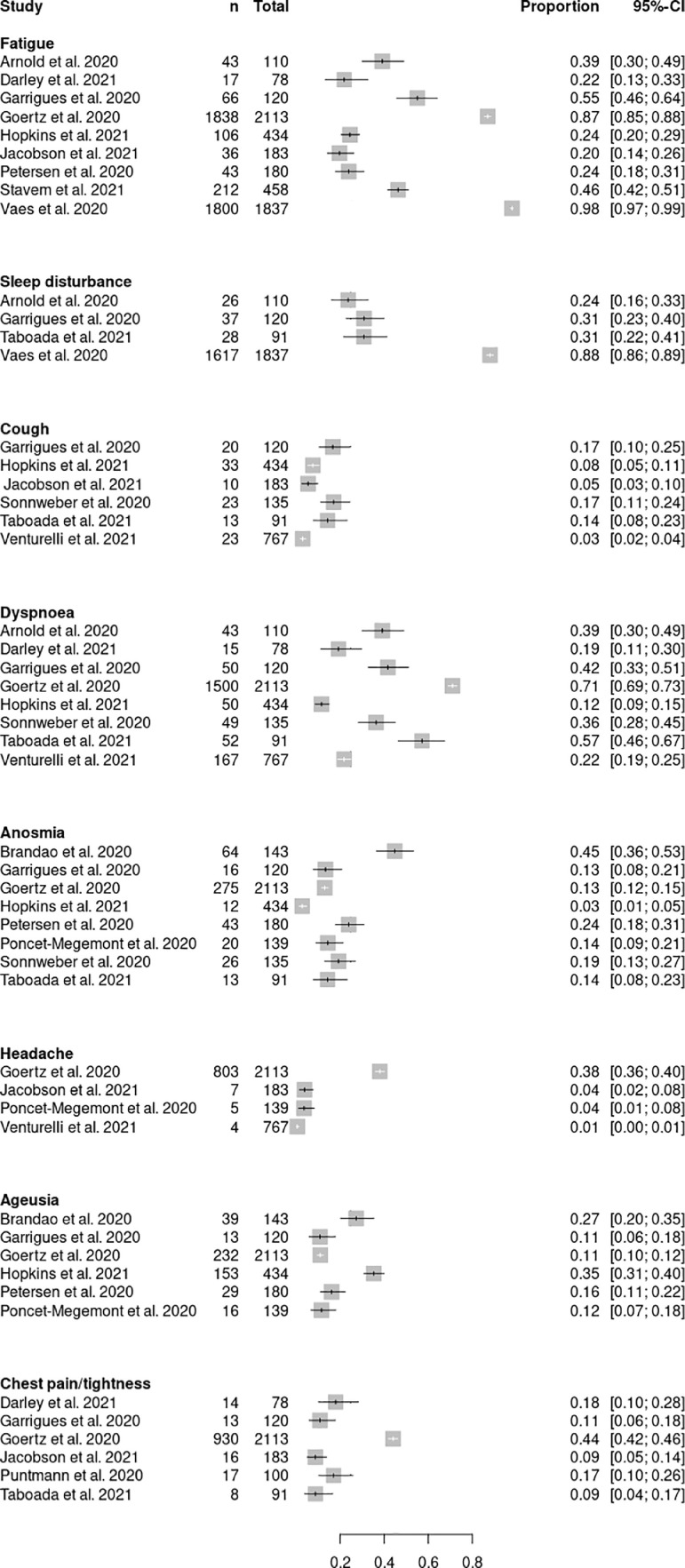
Forest plot of studies describing clinical features in chronic post-COVID syndrome.

All studies reporting the prevalence of clinical features for acute and chronic post-COVID syndrome are shown in Fig. 2, Fig. 3, respectively.

**Fig. 4:**
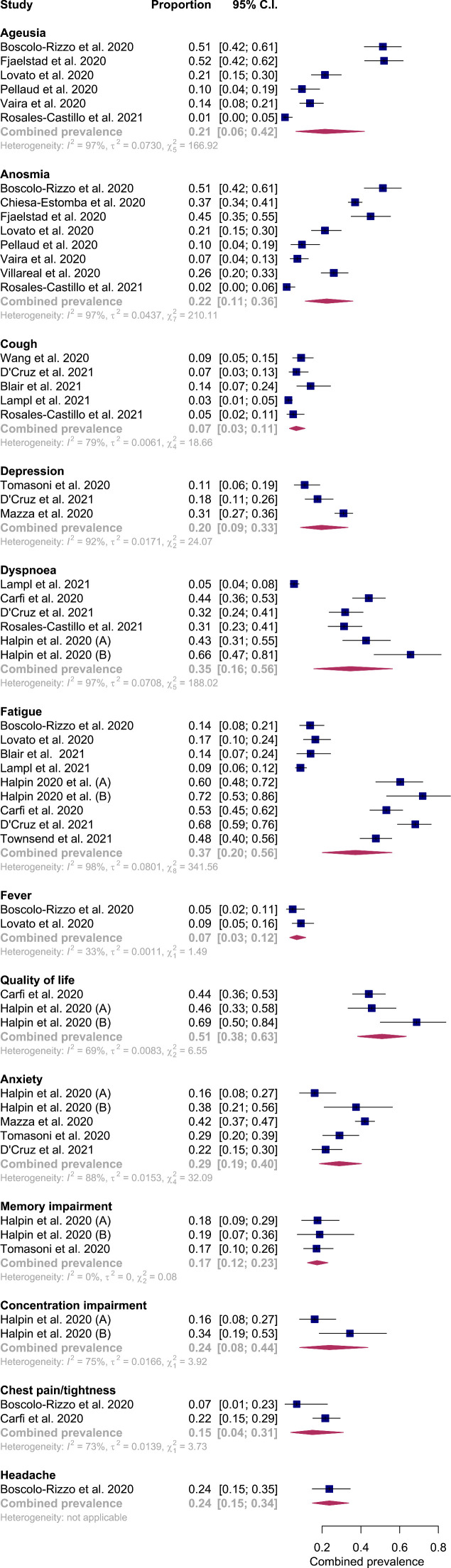
Forest plot of pooled prevalence of clinical features reported in acute post-COVID syndrome.

To meta-analyse the acute post-COVID cohort, seven studies were removed. Two studies study failed to include demographics or characteristics of the included individuals appropriately and was excluded from the sub-group analysis [20,21]. Additionally, Bongiovanni et al. describe potential for inaccurate PCR testing, as such, this study was also excluded from our sub-group analysis [21]. Another study inadequately described follow-up protocols and ascertainment of results [22]. The use of unvalidated questionnaires for retrospective recall in pre-infective functional status and against ‘maximum symptoms’ risks a significant inherent recall bias [23,24]. In addition, strong risks for sample bias from two studies precluded pooled comparisons, including exclusion of hospitalised individuals admitted to intensive care which may underestimate symptom burden [25,26]. The pooled prevalence of clinical features for acute post-COVID syndrome is shown in Fig. 4.

In the acute post-COVID phase, studies reported 13 predominant symptoms allowing for pooled analysis (Fig. 3). The most frequently reported symptoms were fatigue (0·37; 95% CI 0·20-0·56, I^2^ = 98%), dyspnoea (0·35; 95% CI 0·16-0·562, I^2^ = 97%) and anxiety (0·29; 95% CI 0·19-0·40, I^2^ = 88%).

**Fig. 5:**
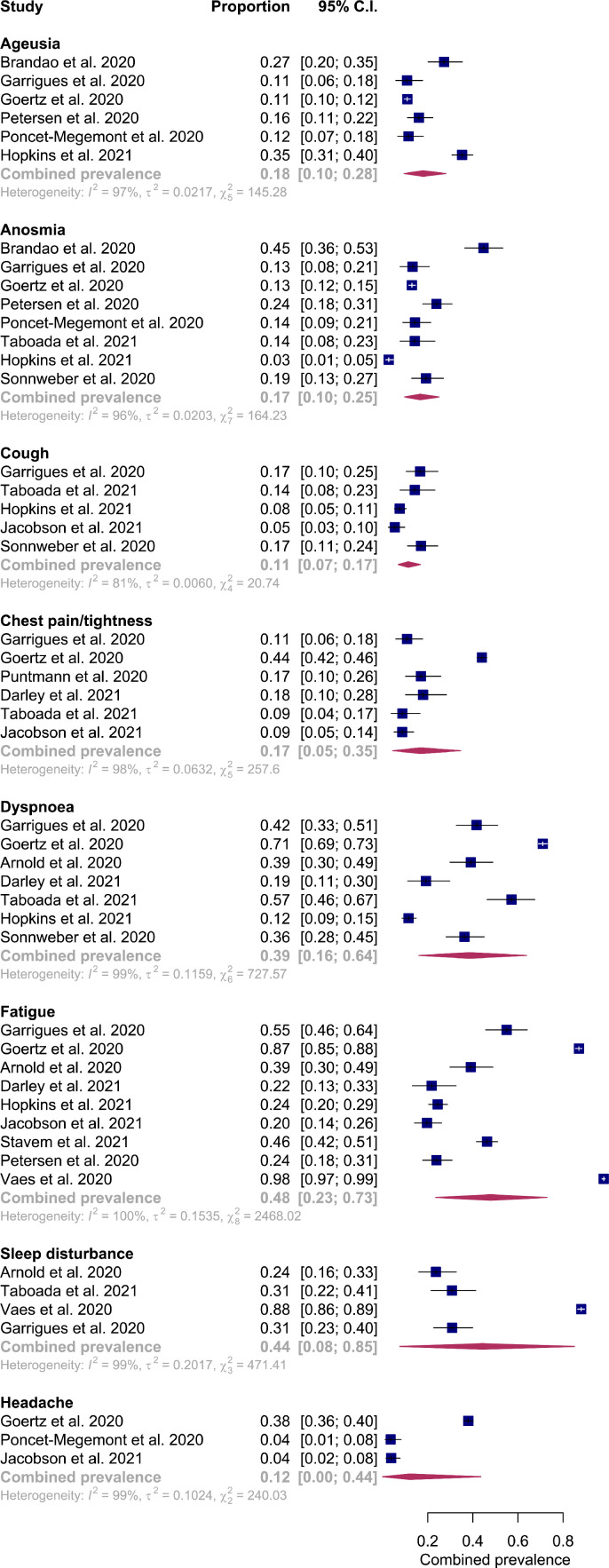
Forest plot of pooled prevalence of clinical features reported in chronic post-COVID syndrome.

The lack of standardisation between enrolment and assessments into the trial precluded one study for the chronic post-COVID syndrome sub-group analysis [27]. Studies reported 8 predominant symptoms allowing for pooled analysis (Fig. 5). Fatigue (0·48; 95% CI 0·23–0·73, I^2^ = 100%), sleep disturbance (0·44; 95%CI 0·08–0·85, I^2^ = 99%), and dyspnoea (0·39; 95% CI 0·16–0·64, I^2^ = 99%) were reported as most prevalent symptoms.

### 3.3 Predictors of post-COVID syndrome

Studies detailing predictors of post-COVID syndrome were limited to five studies. Carvalho-Schneider et al. reported that hospitalisation during the acute infection (odds ratio [OR] 2·9, 95% CI 1·3–6·9) and an age between 40-49 years (OR 15·3, 95% CI 2·8–83·9) were deemed the most significant predictors of developing post-COVID syndrome. The presence of initial symptoms (chest pain, dyspnoea, fever, anosmia, ageusia), gender or number of comorbidities did not predict post-COVID syndrome [19]. However, Goertz et al. contrasts these findings by suggesting that the number of symptoms present during initial infection was most responsible for predicting the number of symptoms at three months [28]. Furthermore, a multivariable analysis adjusting for gender, ethnicity, age, BMI, and hospitalisation status reported that only the presence of fatigue accounted for long-term activity impairment (OR 6·0, 95% CI 1·0–34·9) [29]. Similarly, those with a higher symptom load during the initial infection had greater odds of persistent fatigue [30].

Moreover, the severity of initial infection (i.e., need for critical care admission or invasive ventilation) was associated with patient-reported impairment, although no relationships between age or pre-existing comorbidities and the persistence of post-COVID symptoms were observed [31].

### 3.4 Risk of bias assessment

Twenty-two included studies were deemed to be at high risk of bias; 17 studies were deemed as moderate, and the remaining were considered low risk (Table 1). Frequently, risk of biases surfaced due to lack of control arms, potential effects from confounding variables (e.g., severity of symptoms during acute COVID-19 infection) or a result of strong recall biases given the varied data collection methodology. In addition, limited descriptions of participant recruitment and response rates across studies were noted.

### 3.5 Heterogeneity

Overall, the pooled analyses display significant heterogeneity urging for cautious interpretation of our results. The finding of heterogeneity is partly expected given the pragmatic choice of studies from a range of settings with different study populations (e.g., hospitalised, non-hospitalised, and mixed) with differing co-morbidity demographics (Table 2); differing follow-up timepoints; the varied use of validated and unvalidated questionnaires with significant diverse data collection protocols, such as telephone and face-to-face, are likely to contribute to the observed effects.

In order to explore the sources of substantial heterogeneity, stratification by sampling methodology (previously hospitalised, community, and mixed) was conducted for symptoms displaying an I-squared of over 95% (Appendix 3). Overall, the heterogeneity was lower although, values remained high, particularly in the mixed cohorts (Appendix 3). Further stratification for other factors which we hypothesised to be contributory to the heterogeneity could not be further explored given the insufficient number of papers available for sub-group analysis.

## 4 Discussion

This study suggests that there is a broad range of symptoms that persist beyond the acute phase of COVID-19 in patients with post-COVID syndrome. Fatigue and sleep disturbance were reported to be most common symptoms in acute post-COVID syndrome and fatigue, anxiety and dyspnoea were the most common in chronic post-COVID syndrome. The sizeable prevalence of extra-respiratory (e.g., anosmia) and functional (e.g., fatigue) symptoms illustrates the multi-system burden that post-COVID syndrome imposes upon individuals. Moreover, we also noted that the number of symptoms associated with chronic post-COVID is lower in comparison to the acute post-COVID experience. Lastly, although studies do comment upon specific predictors of post-COVID development, they report varied results, thus hindering clinical application of this knowledge. It seems, however, that severity of initial infection or symptom load during the acute phase of illness is associated with a greater likelihood of continued post-COVID symptomatology.

Given the prevalence of respiratory symptoms in acute COVID infection, the persistence of respiratory symptoms in post-COVID syndrome can be expected. Furthermore, persistent respiratory symptoms are in keeping with previous outbreaks of SARS-CoV which have demonstrated a restrictive pattern of lung function metrics consistent with the resultant muscle weakness six to eight weeks following hospital discharge [32]. Cardiac symptoms were also noted across both acute and chronic post-COVID syndromes. Cardiovascular involvement on cardiac MRI, with myocardial inflammation being the most prevalent abnormality, was observed in 78% of individuals having recovered from acute COVID-19 infection regardless of pre-existing conditions, severity and course of the initial presentation, or presence of cardiac symptoms [33]. The persistence of functional symptoms (e.g., fatigue) could be exacerbated in the context of social distancing and isolation. The pathophysiology of post-COVID syndrome is poorly understood, theories relating to hyperinflammatory state, oxidative stress, cytokine storm, and DNA damage have been hypothesised but on-going research is required for targeting potential treatments [34].

To combat post-COVID syndrome effectively, a multi-faceted approach will be required [2,35]. Current practice consists of following up individuals through self-reported symptoms and remote outpatient clinics. However, the investigations of choice for various symptoms, subsequent monitoring, and need for referral to specialist care has not yet been standardised [2]. The COVID-19 pandemic has seen a marked adoption in health technology. Innovation in technologies have allowed for remote monitoring to take precedent with several trials and evaluations underway [36,37]. One area of future research could see the utilisation of wearable sensors to monitor recovery from COVID-19.

We highlight the limited literature predicting post-COVID syndrome, indicating the need for enhanced surveillance programmes to be employed. Comparisons can be drawn from cancer survivorship in which the development of evidence-based frameworks (e.g., the National Cancer Survivorship Initiative) are deemed essential for the provision of personalised care [11]. Individuals with post-COVID syndrome may experience long lasting effects requiring long lasting support. It has been reported that 15% of individuals were absent from work due to illness at the time of follow-up [18]. It is imperative that this cohort is not forgotten about and broad education is provided to the public to enable better acceptability and understanding.

Policymakers should aim to educate the public and clinicians concerning post-COVID syndrome, thus recognising it as a legitimate health condition [11]. There is demand for a tailored approach towards recovery to pre-empt issues in advance. To achieve this, and given the scarcity of current data, there is an urgent need to drive recruitment into COVID-19 trials to improve our understanding and better identify predictors of symptom clusters [38].

Despite the importance of our work, a series of limitations are to be mentioned. Whilst the broad inclusion of studies, including those deemed at high risk of bias, in our analysis resulted in significant heterogeneity (with high I-squared values), it allowed for a broad insight into the prevalence and predictors of post-COVID syndrome based on the current literature in a condition with a limited but growing evidence base. Studies reported a mixture of cohort sampling (previously hospitalised, community, and mixed), follow-up timepoints, and data collection protocols which likely contributed to the existing heterogeneity. Further sub-group analyses assisted in providing insight into this heterogeneity, with mixed cohorts displaying large I-squared values (Appendix 3). However, given the pragmatic nature of study inclusion, this was expected. Calls to incorporate post-COVID sufferers’ perceptions within its evolving definition suggests the grouping of these cohorts within our analysis may assist in depicting the overall disease burden. Furthermore, the majority of published literature excludes low-income countries, an important omission in the midst of a global pandemic given available resources distinctly tailor potential available strategies for surveillance and treatment. Moreover, included cross-sectional studies consisted of small sample sizes; were single-centre; and involved questionnaires requiring retrospective recall of symptoms resulting in potential for recall bias and subjective assessment [18,19,[39], [40], [41], [42]]. Indeed, this methodology fails to capture the evolution of symptoms over time. In one study, attempts to overcome this through multiple phone calls at various time points was made; however, only a small proportion of participants responded to repeated calling [41]. A further study identified participants through long-COVID groups on Facebook, and eligible individuals were invited to join a registry and then respond to questionnaires [28]. However, this relied on technological literacy, risking selection and ascertainment bias. Qualitative experiences were measured on individuals that did not require hospitalisation for either the acute COVID-19 infection nor for post-COVID symptoms; participants were predominantly female and under-representative of BAME communities, reducing overall generalisability [43]. Lastly, the literature on predictors for post-COVID syndrome remains limited with one study excluding severe (intensive care) COVID-19 cases and including participants younger in age, many of whom were healthcare professionals, limiting generalisability to the public [19].

Varied terminology (e.g., ‘long COVID’, and ‘post-COVID syndrome’) have contributed to heterogenous research; given the adoption of the latter by NICE guidelines, widespread adoption of the term ‘post-COVID syndrome’ is required to aid homogenisation of future symptom data, allowing predictors to be accurately described [12]. The introduction of clinical codes for chronic post-COVID syndrome may aid identification of cases from administrative clinical datasets [44]. Moreover, prospectively designed trials with appropriate control arms are required (including low- and middle-income countries) to establish relationships between post-COVID syndrome and i) age, particularly as several studies excluded elderly populations which are most at risk of severe symptoms; ii) ethnicity status; and iii) characteristics and severity of initial acute infection (e.g., requirement of intensive care, need for supplemental oxygen).

In conclusion, the applicability of current knowledge on post-COVID syndrome is limited by the quality of available data, a result of the flaws in data capture and interpretation, as demonstrated in the uncertainty of our meta-analysis and there is need for global collaboration to further understand the prevalence, clinical characteristics, and prognosis of this novel disease. Clinicians, policy makers, and researchers must focus on understanding the impact of this condition on individuals and society.

## Declaration of Competing Interest

None.

## Appendix Supplementary materials

Image, application 1
