# Supplementary material for: Characteristics and predictors of acute and chronic post-COVID syndrome: A systematic review and meta-analysis
Source: eClinicalMedicine. 2021 May 24;36:100899. doi: 10.1016/j.eclinm.2021.100899 (PMC8141371; doi:10.1016/j.eclinm.2021.100899)

Appendix 1: Search Strategy

# Ovid Medline

1 (long covid or long-haul covid).mp.

2 post-covid syndrome.mp.

3 exp coronaviridae/ or exp coronavirus/

4 (coronavirus or corona virus or coronavirinae).mp.

5 (covid-19 or 2019-nCoV or SARS-CoV or SARS-COV-2 or MERS-CoV).mp.

6 or/3-5

7 ((post-virus or post-viral or persisten$ or ongoing or fail$ or incomplete) adj3 recover$).mp.

8 ((post-virus or post-viral or persist$ or ongoing) adj3 (illness or symptom$ or fatigue$ or unwell)).mp.

9 or/7-8

10 6 and 9

11 1 or 2 or 10

12 (chronic adj1 (coronavirus or corona virus or coronavirinae or covid-19 or 2019-nCoV or SARS-CoV or SARS-COV-2 or MERS-CoV)).mp.

13 11 or 12

14 ((long-haul$ or lasting illness) adj1 (coronavirus or corona virus or coronavirinae or covid-19 or 2019-nCoV or SARS-CoV or SARS-COV-2 or MERS-CoV)).mp.

15 13 or 14

# EMBASE

1 (long covid or long-haul covid).mp

2 post-covid syndrome.mp.

3 exp coronaviridae/

4 (coronavirus or corona virus or coronavirinae).mp.

5 (covid-19 or 2019-nCoV or SARS-CoV or SARS-COV-2 or MERS-CoV).mp.

6 or/3-5

7 ((post-virus or post-viral or persisten$ or ongoing or fail$ or incomplete) adj3 recover$).mp.

8 ((post-virus or post-viral or persist$ or ongoing) adj3 (illness or symptom$ or fatigue$ or unwell)).mp.

9 or/7-8

10 6 and 9

11 1 or 2 or 10

12 (chronic adj1 (coronavirus or corona virus or coronavirinae or covid-19 or 2019-nCoV or SARS-CoV or SARS-COV-2 or MERS-CoV)).mp.

13 11 or 12

14 ((long-haul$ or lasting illness) adj1 (coronavirus or corona virus or coronavirinae or covid-19 or 2019-nCoV or SARS-CoV or SARS-COV-2 or MERS-CoV)).mp.

15 13 or 14

# PsychINFO

1 (long covid or long-haul covid).mp.

2 post-covid syndrome.mp.

3 exp coronavirus/

4 (coronavirus or corona virus or coronavirinae).mp.

5 (covid-19 or 2019-nCoV or SARS-CoV or SARS-COV-2 or MERS-CoV).mp.

6 or/3-5

7 ((post-virus or post-viral or persisten$ or ongoing or fail$ or incomplete) adj3 recover$).mp.

8 ((post-virus or post-viral or persist$ or ongoing) adj3 (illness or symptom$ or fatigue$ or unwell)).mp.

9 or/7-8

10 6 and 9

11 1 or 2 or 10

12 (chronic adj1 (coronavirus or corona virus or coronavirinae or covid-19 or 2019-nCoV or SARS-CoV or SARS-COV-2 or MERS-CoV)).mp.

13 ((long-haul$ or lasting illness) adj1 (coronavirus or corona virus or coronavirinae or covid-19 or 2019-nCoV or SARS-CoV or SARS-COV-2 or MERS-CoV)).mp.

Appendix 2: Statistical analysis

A meta-analysis of proportions was conducted using the metaprop command within the meta package in RStudio version 3.6.3 (R Studio, Boston, MA, USA)^1^.

In order to allow the observed proportions to follow a normal distribution and stabilise the variance, the Freeman-Tukey double arcsine transformation was applied as follows^2^:

$$arcsin\sqrt{\frac{x}{n+1}}+arcsin\sqrt{\frac{x+1}{n+1}}$$

(1)

Heterogeneity was assessed using Higgin’s I^2^ which measures percentage of total variation across trials^3^. We considered a value less than 30% as low heterogeneity, between 30-60% moderate, and over 60% as high. This was calculated as follows:

$$I^{2}=100\%\times\frac{(Q-df)}{Q}$$

(2)

where: *Q* = Cochran’s heterogeneity statistic

*df* = degrees of freedom

Effect size was calculated using a random effects model due to the likely presence of between-study variance and estimated using the DerSimonian and Laird method^4^. The pooled estimate and 95% confidence limits on the transformed scale were back-transformed to provide the pooled summary estimate and confidence limits calculated through the Clopper-Pearson interval^5^.

Back-transformation was achieved as per Miller, 1978^6^:

$$p= \frac{1}{2}\left[ 1-sgn(\cos t)\sqrt{\left[ 1-{(\sin t+ \frac{\sin t-\frac{1}{\sin t}}{n^{'}})}^{2} \right]} \right]$$

(3)

where: *t* = transformed value

*sgn* = sign operator whereby $\mathrm{sgn} x:=\left\{ \begin{aligned} -1 if x<0, \\ 0 if x=0, \\ 1 if x>0. \end{aligned} \right.$

*n’* = harmonic mean of individual sample sizes

References:

1. RDocumentation. metaprop: Meta-analysis of single proportions. <https://www.rdocumentation.org/packages/meta/versions/4.9-6/topics/metaprop>.

2. Freeman MF, Tukey JW. Transformations Related to the Angular and the Square Root. *The Annals of Mathematical Statistics* 1950; **21**(4): 607-11, 5.

3. Higgins JPT, Thompson SG, Deeks JJ, Altman DG. Measuring inconsistency in meta-analyses. *BMJ* 2003; **327**(7414): 557-60.

4. DerSimonian R, Laird N. Meta-analysis in clinical trials. *Control Clin Trials* 1986; **7**(3): 177-88.

5. Newcombe RG. Two-sided confidence intervals for the single proportion: comparison of seven methods. *Stat Med* 1998; **17**(8): 857-72.

6. Miller JJ. The Inverse of the Freeman – Tukey Double Arcsine Transformation. *The American Statistician* 1978; **32**(4): 138-.

Appendix 3: Sub-group meta-analysis

Acute Post-COVID syndrome

Figure 1: Ageusia


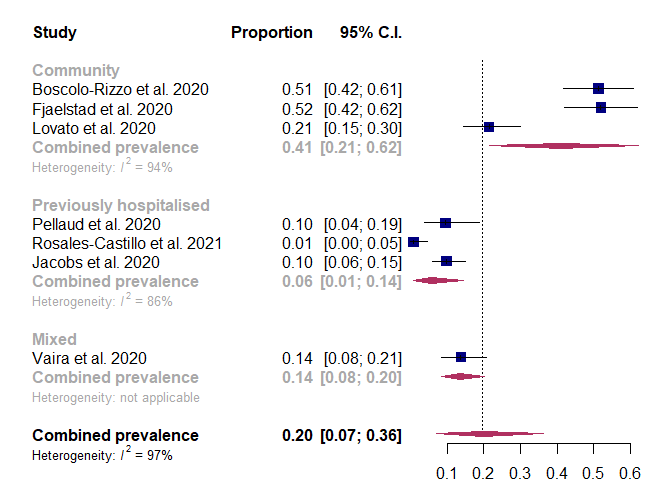


Figure 2: Anosmia


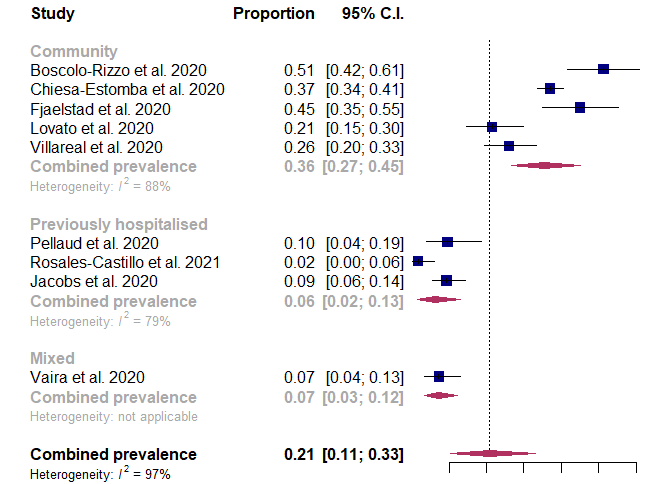


Figure 3: Cough


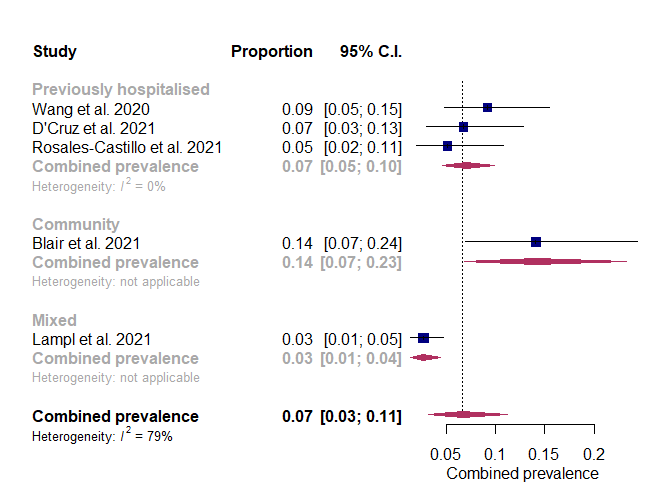


Figure 4: Depression


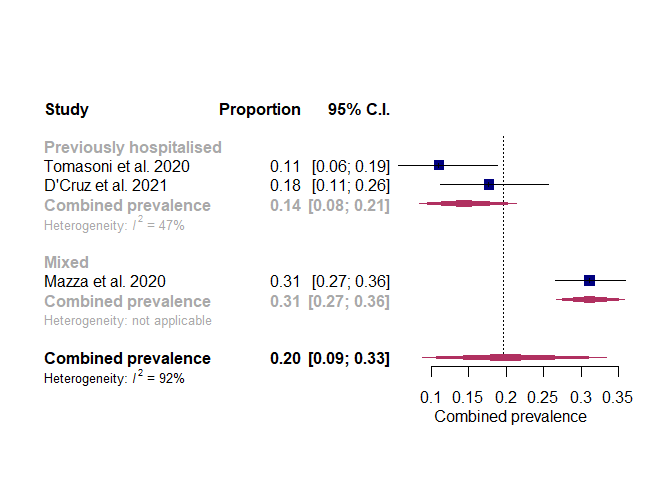


Figure 5: Dyspnoea


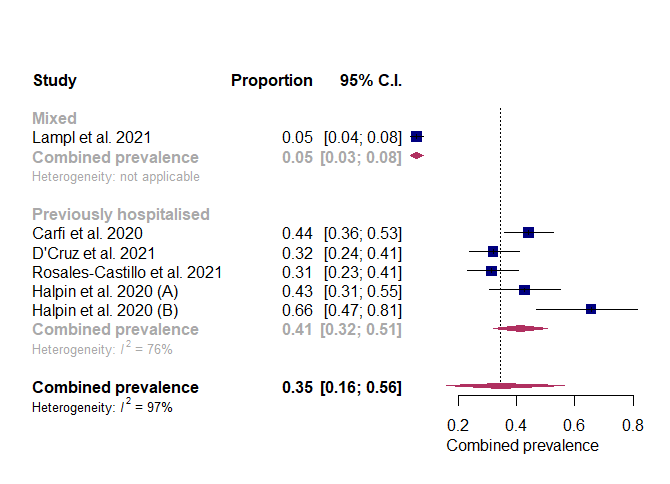


Figure 6: Fatigue


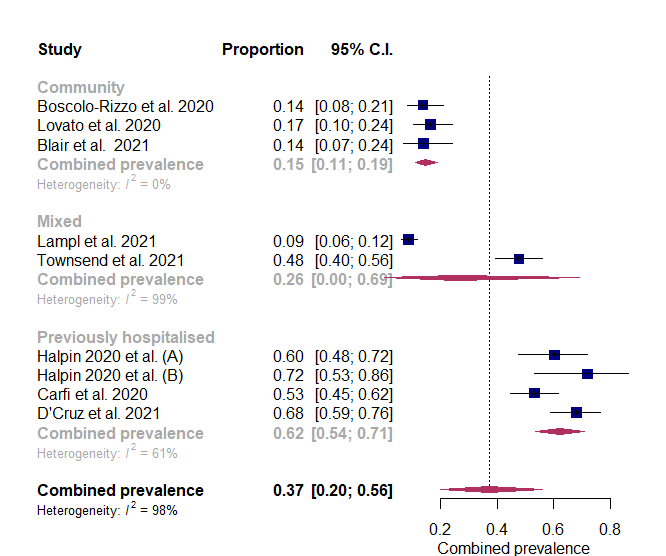


# Chronic Post-COVID Syndrome

Figure 7: Ageusia


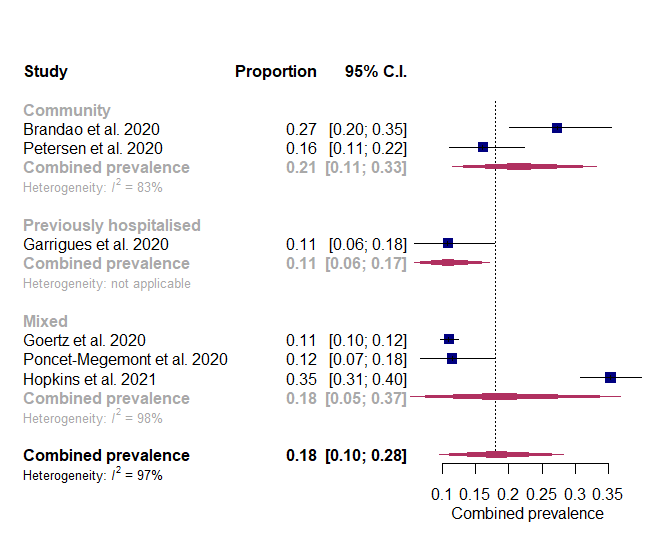


Figure 8: Anosmia


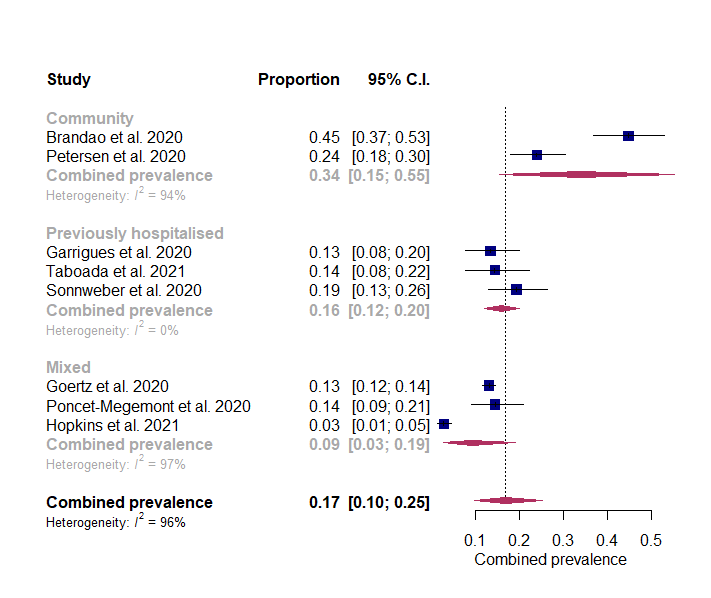


Figure 9: Cough


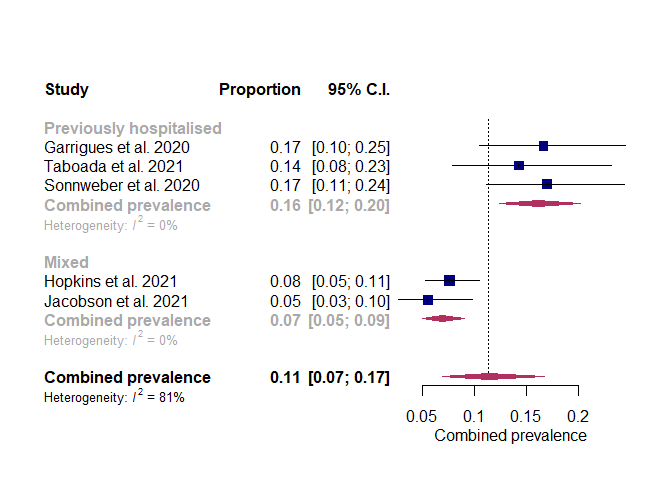


Figure 10: chest pain/tightness


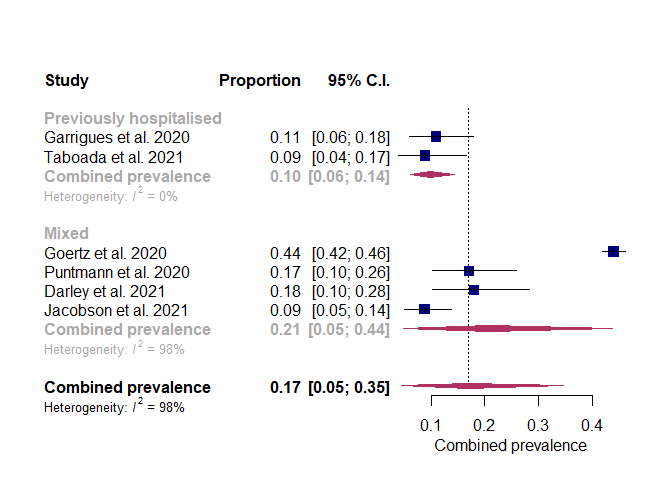


Figure 11: dyspnoea


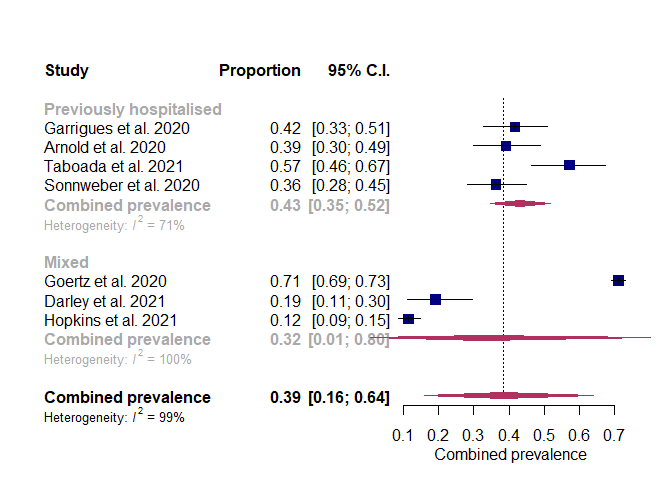


Figure 12: fatigue


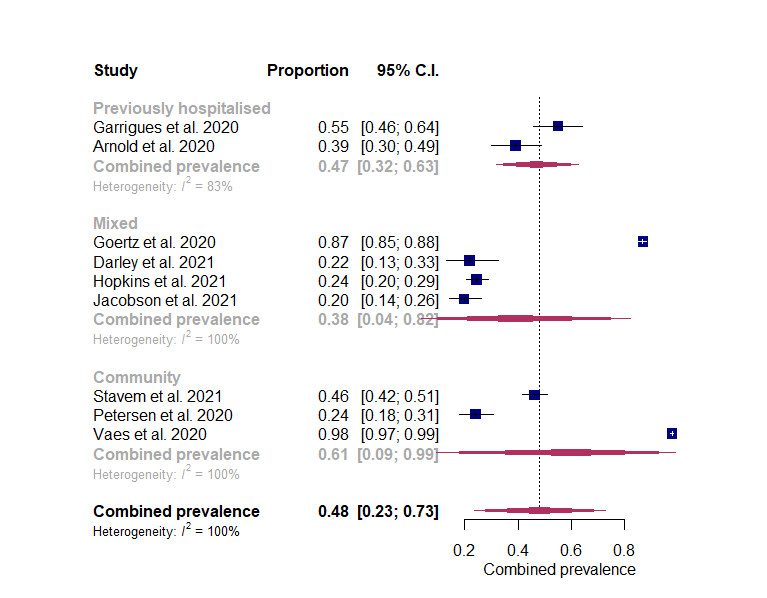


Figure 13: headache


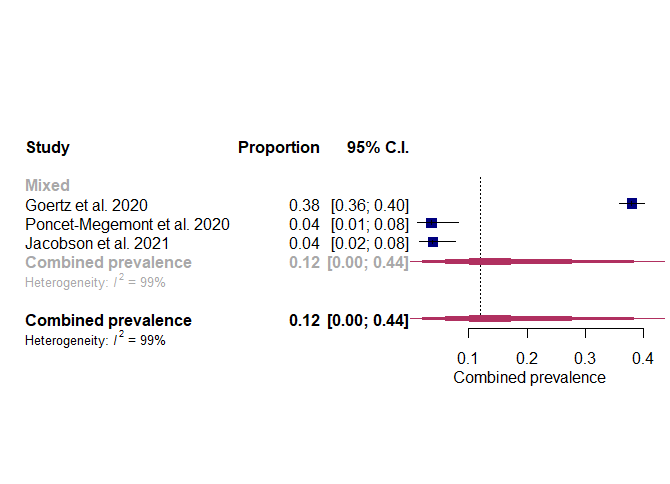


Figure 14: sleep disturbance


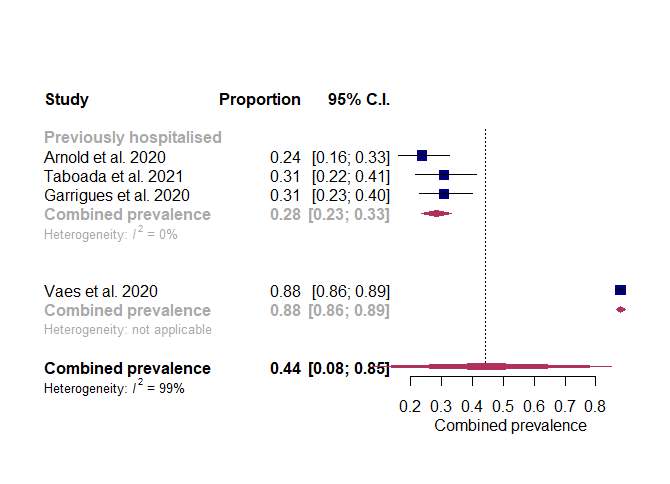

Supplement: Supplementary file 1 [file mmc1.docx]
